# Supplementary material for: Perfect flat band with chirality and charge ordering out of strong spin-orbit interaction
Source: Nat Commun. 2022 Feb 1;13:579. doi: 10.1038/s41467-022-28132-y (PMC8807784; doi:10.1038/s41467-022-28132-y)
Supplement: Supplementary file 1 — Supplementary Information [file 41467_2022_28132_MOESM1_ESM.pdf]

Supplementary Information for "Perfect flat band with chirality and charge ordering out of strong spin-orbit interaction"

Hiroki Nakai* and Chisa Hotta†
Department of Basic Science, University of Tokyo, Meguro-ku, Tokyo 153-8902, Japan

Content :

A. Atomic levels of 5d materials
B. Estimation of material parameters
C. Slater-Koster parameters
D. Noninteracting band structures
E. Details of the mean-field solution
F. Spinor line graph theory for the kagome lattice and some comments
G. Construction of many body flat band state

A. Atomic levels of 5d materials

We consider atomic levels of 5d electrons within mind W-atoms of CsW_2O_6 forming a pyrochlore lattice. The W-atom is surrounded by six oxygens forming an octahedron. The octahedron is slightly compressed and distorted (see Supplementary Figure 1(a)). The degree of distortion is measured by an angle θ, where we take a local z-axis of the octahedron with trigonal symmetry as those pointing perpendicular to the faced triangles. From a single crystal XRD data in Ref.[1], we evaluate the angle $\theta = 55.71°$ for 250K (phase I), which is slightly larger than $\theta_0 = 54.74° = \arccos(1/\sqrt{3})$ of the undistorted octahedron.

The Hamiltonian for the present trigonal crystal field $\mathcal{H}_{\rm CF}$ is expanded in terms of spherical harmonics $Y_n^m(\theta, 0)$ as

$$\begin{aligned}\mathcal{H}_{\rm CF} &= Ze^2\frac{\langle r^2\rangle}{R^3}\frac{24\pi}{5}Y_2^0\boldsymbol{Q}_{20} + Ze^2\frac{\langle r^4\rangle}{R^5}\frac{8\pi}{3}\left(Y_4^0\boldsymbol{Q}_{40} + Y_4^3\boldsymbol{Q}_{43}\right)\\ &= 10Dq\Big(\frac{8\pi}{5}\left(Y_4^0\boldsymbol{Q}_{40}+Y_4^3\boldsymbol{Q}_{43}\right) + \frac{24\pi}{5}\kappa Y_2^0\boldsymbol{Q}_{20}\Big) \qquad \text{(S1)}\end{aligned}$$

where $\kappa = R^2\langle r^2\rangle/\langle r^4\rangle$ with R being the metal(W)-ligand(O) distance, and $10Dq = 5Ze^2\langle r^4\rangle/3R^5$ is the energy splitting between t_{2g} and e_g levels for an undistorted octahedron. We take the basis set as $\big(|xy\rangle, |yz\rangle, |zx\rangle, |x^2-y^2\rangle, |3z^2-r^2\rangle\big)$ for the above mentioned z-axis in the (1,1,1)-direction, and the matrix representations are given as

$$\begin{aligned}\boldsymbol{Q}_{40} &= \frac{-1}{14\sqrt{\pi}}\,\mathrm{diag}\Big(-1,\,4,\,4,-1,-6\Big)\\ \boldsymbol{Q}_{20} &= \frac{-\sqrt{5}}{14\sqrt{\pi}}\,\mathrm{diag}\Big(2,-1,-1,\,2,-2\Big)\\ \boldsymbol{Q}_{43} &= \frac{-\sqrt{35}}{14\sqrt{\pi}}\begin{pmatrix}0 & -1 & & & \\ -1 & 0 & & & \\ & & 0 & 1 & \\ & & 1 & 0 & \\ & & & & 0\end{pmatrix}. \qquad \text{(S2)}\end{aligned}$$

We introduce a set of parameters that include $\boldsymbol{Q}$ and $Y_n^m(\theta,0)$ following Ref.[2] as

$$\begin{aligned} a &= a_2 + a_4,\\ a_2 &= \frac{108\sqrt{5\pi}}{175}\kappa\, Y_2^0 = \frac{27}{35}\kappa(3\cos^2\theta - 1),\\ a_4 &= -\frac{4\sqrt{\pi}}{7}Y_4^0 = -\frac{3}{2}\left(\frac{5}{2}\cos^4\theta - \frac{15}{7}\cos^2\theta + \frac{3}{14}\right),\\ b &= -8\sqrt{\frac{\pi}{35}}Y_4^3 = 3\sin^3\theta\cos\theta. \qquad \text{(S3)}\end{aligned}$$

By diagonalizing Eq.(S1) we first obtain five energy levels in a trigonal crystal field, e_g^σ doublet, e_g^π doublet, and a_{1g}, as shown in Supplementary Figure 1(b), whose eigenstates are given as

$$\begin{aligned} |a_{1g}\rangle &= |3z^2-r^2\rangle & L_z^{\mathit{eff}} &= 0,\\ |e_{g1}^\pi\rangle &= (i|g_1\rangle - |g_2\rangle)/\sqrt{2} & L_z^{\mathit{eff}} &\sim +1,\\ |e_{g2}^\pi\rangle &= (i|g_1\rangle + |g_2\rangle)/\sqrt{2} & L_z^{\mathit{eff}} &\sim -1,\\ |g_1\rangle &= -\cos\frac{\alpha}{2}|xy\rangle + \sin\frac{\alpha}{2}|yz\rangle, & &\\ |g_2\rangle &= \cos\frac{\alpha}{2}|x^2-y^2\rangle + \sin\frac{\alpha}{2}|zx\rangle, & &\\ \cos\alpha &= \frac{a}{\sqrt{a^2+b^2}}. & & \qquad \text{(S4)}\end{aligned}$$

The effective angular momentum L_z^{eff} deviates from ±1 for e_{g1}^π because of the trigonal distortion and from the effect of other metal ions, which are reflected in the parameter a_2 as we discuss shortly. The energy levels are obtained as

$$\begin{aligned} E_{a_{1g}} &= \frac{-18a_4 + 10a_2}{15},\\ E_{e_g^{\pi/\sigma}} &= \frac{9a_4 - 5a_2}{30} \mp \frac{1}{2}\sqrt{a^2+b^2}, \qquad \text{(S5)}\end{aligned}$$

where $-/+$ are for $e_g^\pi/e_{g1,2}^\sigma$ and we denote the energy difference between lower two levels as $\Delta_1 = E_{e^\pi_{g1,2}} - E_{a_{1g}}$, which is positive for $\theta > \theta_0$ and negative for $\theta < \theta_0$. For a regular octahedron ($\theta = \theta_0$), $\Delta_1 = 0$ and we find $\cos\frac{\alpha}{2} = \frac{2}{\sqrt{6}}$ and $\sin\frac{\alpha}{2} = \frac{1}{\sqrt{3}}$. The two energy scales, $10Dq$ and κ, together with θ determines the value of Δ_1.

The parameter κ which is the ratio of coefficients between the two diagonal terms in Eq.(S1) is roughly evaluated by considering the integral $\langle r^n\rangle = \int_0^\infty r^n R_d^2(r) r^2 dr$ and $R_d(r) \sim r^3\exp(-z^*r/4a_B)$, where $R_d(r) \sim r^{n^*-1}\exp(-z^*r/(n^*a_B))$, z^* is the effective nuclear charge considering a screening effect, $n^* \sim 4(3)$ for $5d(3d)$ electrons, and a_B is a Bohr radius. For CsW_2O_6,

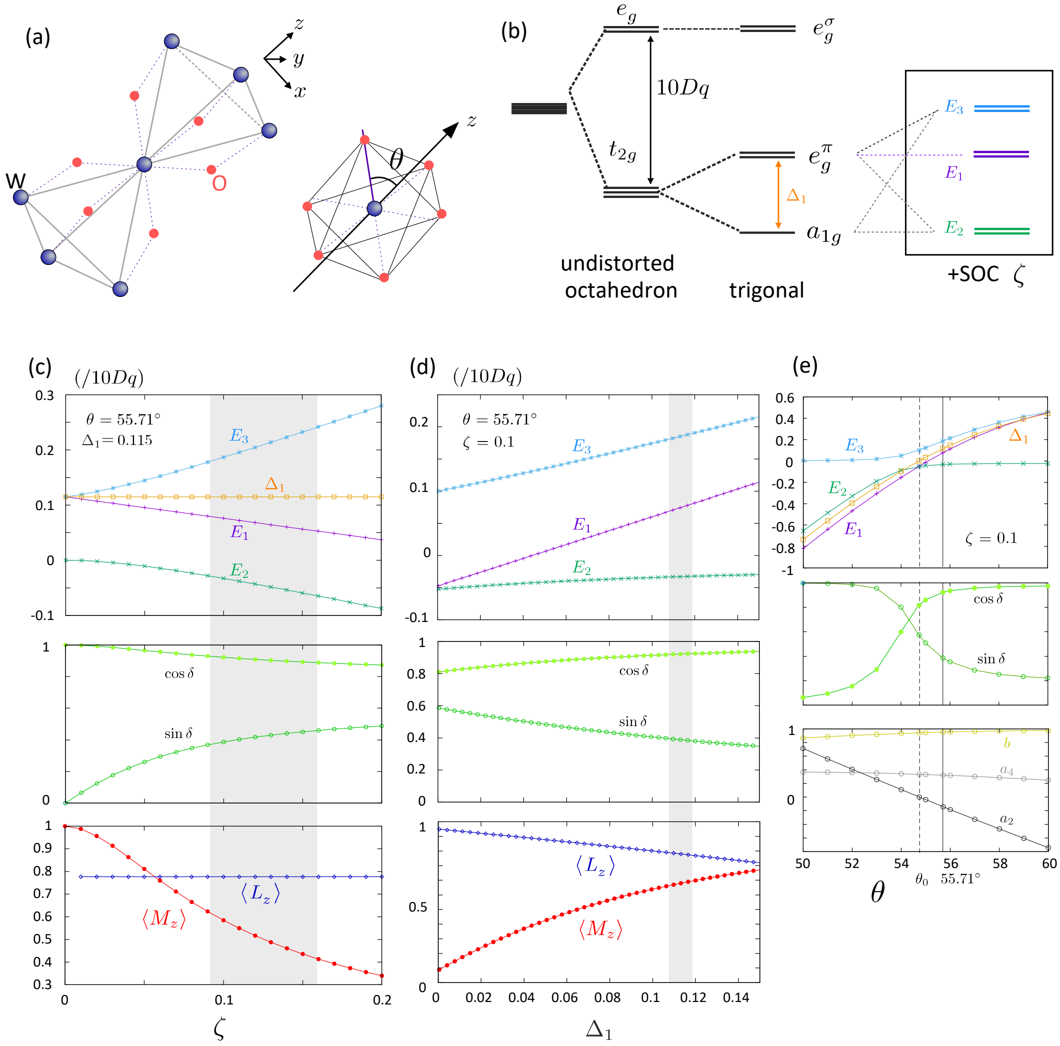

Supplementary Figure 1. (a) Pyrochlore lattice consisting of W-atoms and the surrounding oxigen octahedron(red circle). (b) Crystal field splitting including the spin-orbit coupling ζ. (c-e) Energy levels $E_1 \sim E_3$, weights of a_{1g} and e_g^π orbitals to E_2 doublets ($\cos\delta$ and $\sin\delta$), and the angular moment L_z^{eff} and magnetic moment $\langle M_z \rangle$ evaluated for the lowest doublet. We plot them as functions of ζ, crystal field splitting Δ_1 and θ.

we take the W-O distance $R \sim 1.95$ Å and the Slater's rule $z^* = 6$ ($z = 74$ for W-atom), and find $\kappa = 0.923$.

However, in reality, this evaluation does not straightforwardly reproduce the experimental observation nor a first-principles band structural calculation. For example, for the materials listed in Supplementary Table I we show the values of $\kappa_{\rm mat}$ which reproduce Δ_1 obtained in experiments using $\mathcal{H}_{\rm CF}$ to compare with $\kappa_{sl} = (R/a_B)^2(z^*)^2/528$ from the evaluation in the previous paragraph using Ir^{4+} ($z^* = 7.6$),Os^{3+} ($z^* = 7.3$) and $W^{5.5+}$ ($z^* = 6$). Although they take the same orders, it is difficult to accurately determine κ and accordingly Δ_1 from the Slater's rule and above mentioned formula.

This shall be because Eq.(S1) is considering an isolated octahedron, neglecting the effect of neighboring metal atoms. As discussed in Ref.[2], taking into account the contribution to the crystal field from these atoms largely modifies the a_2 term which basically increases Δ_1. Here, we determine $a_2 = -0.142$ to reproduce $\Delta_1 \sim 0.23$eV anticipated from the band structural calculation as we explain in Supplementary B.

Next, we introduce a spin-orbit coupling ζ and diagonalize

$$\mathcal{H} = \zeta \boldsymbol{l} \cdot \boldsymbol{s} + \mathcal{H}_{\rm CF}. \tag{S6}$$

Here, we exclude the upper e_g^σ doublet which is much higher in energy than the lower three levels. The remaining a_{1g} and e_g^π levels are rearranged into three doublets

Supplementary Table I. Data of materials taken from Refs.[3–6] $\kappa = (R/a_B)^2(z^*)^2/528$ is evaluated from the effective charge following Slater's rule. $\kappa_{\rm mat}$ is the value we derived to reproduce the experimental Δ_1 obtained from Eq.(S1).

	$Eu_2Ir_2O_7$	Na_2IrO_3	$Cd_2Os_2O_7$	CsW_2O_6
$10Dq$ [eV]	3.5	3.3	—	2.0
Δ_1 [eV]	0.45	0.075	0.0966	0.4
θ [deg]	60.64	57.96	56.64	55.71
R [Å]	2.03	2.06	1.926	1.95
ζ [eV]	0.5	0.43	0.332	0.2-0.3
$\kappa_{\rm Sl}$	1.6	1.65	1.33	0.923
$\kappa_{\rm mat}$	1.5	0.87	1.05	3.85
$a_{2\rm mat}$	-0.345	-0.198	-0.095	-0.142

with energy E_1, E_2 and E_3 as

$$\begin{aligned} E_1 &= 2(\Delta_1 - \Delta), \\ E_2 &= \Delta - \sqrt{\Delta^2 + \xi^2}, \\ E_3 &= \Delta + \sqrt{\Delta^2 + \xi^2}, \\ \Delta &= \frac{\Delta_1}{2} + \frac{1 + 3\cos\alpha}{8}\zeta, \quad \xi = \zeta\sqrt{\frac{3}{2}}\sin\frac{\alpha}{2}, \end{aligned} \tag{S7}$$

where the corresponding wave functions $|\phi_\sigma^{(i)}\rangle$ for doublets $i = 1 \sim 3$ are the combination of t_{2g} levels and electron spins $\sigma =\uparrow, \downarrow$ as

$$\begin{aligned} &|\phi_\uparrow^{(1)}\rangle = |e_{g1}^\pi; \uparrow\rangle, \ \ |\phi_\downarrow^{(1)}\rangle = |e_{g2}^\pi; \downarrow\rangle, \ \ (J_{\mathit{eff}} \sim \frac{3}{2}, \ J_{\mathit{eff}}^z \sim \pm\frac{3}{2}) \\ &|\phi_\uparrow^{(2)}\rangle = \cos\delta |a_{1g}; \uparrow\rangle + \sin\delta |e_{g1}^\pi; \downarrow\rangle, \\ &|\phi_\downarrow^{(2)}\rangle = \cos\delta |a_{1g}; \downarrow\rangle + \sin\delta |e_{g2}^\pi; \uparrow\rangle, \ (J_{\mathit{eff}} \sim \frac{3}{2}, \ J_{\mathit{eff}}^z \sim \pm\frac{1}{2}) \\ &|\phi_\uparrow^{(3)}\rangle = \sin\delta |a_{1g}; \uparrow\rangle - \cos\delta |e_{g1}^\pi; \downarrow\rangle, \\ &|\phi_\downarrow^{(3)}\rangle = \sin\delta |a_{1g}; \downarrow\rangle - \cos\delta |e_{g2}^\pi; \uparrow\rangle, \ (J_{\mathit{eff}} \sim \frac{1}{2}, \ J_{\mathit{eff}}^z \sim \pm\frac{1}{2}) \\ &\tan 2\delta = \frac{\xi}{\Delta}. \end{aligned} \tag{S8}$$

For a finite trigonal distortion $\theta \neq \theta_0$, these doublets are not exact eigenstates of effective angular momentum J_{eff}. However, assigning quantum numbers J_{eff} and J_{eff}^z to these doublets is convenient to understand the major contribution of angular momentums and types of orbitals to these doublets and is commonly used [3,7]. For E_2 doublets, we evaluate the actual value for $\theta = 55.71°$, and find that $L_z^{\mathit{eff}} \sim 0.8$, which gives $J_{\mathit{eff}} \sim 0.3$ and 1.3.

We are interested in the case where the E_2-doublet becomes the lowest in energy where maximally one electron occupies this level. These doublets carry magnetic moment coming originally from the $J_{\mathit{eff}} = 3/2$ quartet, and we can evaluate the net magnetic moment as $M = 2S - L^{\mathit{eff}}$ where L^{eff} is the effective momentum of t_{2g} orbitals. The magnetic moment of the E_2-doublet is obtained as

$$|\langle M_z\rangle| = \cos^2\delta - \sin^2\delta\left(1 + \left(2\cos^2\frac{\alpha}{2} - \sin^2\frac{\alpha}{2}\right)\right), \tag{S9}$$

where $L_z^{\mathit{eff}} = 2\cos^2\frac{\alpha}{2} - \sin^2\frac{\alpha}{2}$ becomes ± 1 for the undistorted octahedron.

We plot in Supplementary Figures 1(c) and 1(d) the energy levels $E_1 \sim E_3$, the weight of a_{1g} and e_g^π orbitals ($\cos\delta$ and $\sin\delta$), and $\langle M_z\rangle$ (for the lowest energy level) and L_z^{eff} as a function of ζ and Δ_1, respectively. We consider that CsW_2O_6 has $\Delta_1 = 0.115$ $(10Dq)$, which is determined from $\mathcal{H}_{\rm CF}$, namely from Eqs.(S3-S5). Here, we evaluated the values of a_4 and b based on $\theta = 55.71°$ and a_2 based on the first principles calculation (see Supplementary B). With increasing ζ, the mixing of a_{1g} and e_g^π orbitals becomes larger and $\langle M_z\rangle$ becomes smaller. The energy splitting $E_1 - E_2$ increases with Δ_1, which can be understood from Supplementary Figure 1(e) as the increase of θ, namely from the effect of crystal field. One can also see that the large amplitude of negative a_2 is important to have Δ_1 large. The effect of crystal field from the other metal ions is known to increase the amplitude of a_2 by orders of magnitude[2].

Here, we take the crystal field splitting between the e_g and t_{2g} orbitals, $10Dq \sim 2$eV, as an energy unit (see Supplementary B). Since the SOC of W-ions are considered to be more than half of those of Iridates, namely $200 - 300$meV, we consider $\zeta \sim 0.1 - 0.15(10Dq)$. Then we expect $E_1 - E_2 \sim 0.1(10Dq) = 0.2$eV from Supplementary Figure 1(c), which is enough large compared to the transfer integral $t \sim 0.06$ eV evaluated separately from the band structural calculation (see Supplementary B). Since we are dealing with the bottom energy level with no band dispersion, our approximation of taking only the lowest E_2-doublet is verified.

B. Estimation of material parameters

We determine the model parameters referring to the first principles calculation shown in Supplementary Figure 2, and using the formulation in Supplementary A. The parameter known *a priori* from the experiments is $\theta = 55.71°$, which determines a_4 and b, while a_2 and $10Dq$ are unknown. The uncertainty of a_2 is because of the difficulty of determining κ and also the lack of information from the crystal field from other ions. We thus need to determine these two parameters from Supplementary Figure 2.

The first-principles DFT calculation in Supplementary Figure 2 was performed by Yamakawa as a series of calculations in Ref.[1] in the right panel of Fig. 3 with four primitive cells and the same structural parameter. (In Ref.[1] the one in Supplementary Figure 2 was not explicitly presented). In Supplementary Figure 2(a) we highlighted three energy windows in which e_g^σ, e_g^π and a_{1g} levels have dominant contributions to the energy bands. These regions are set based on the information of the

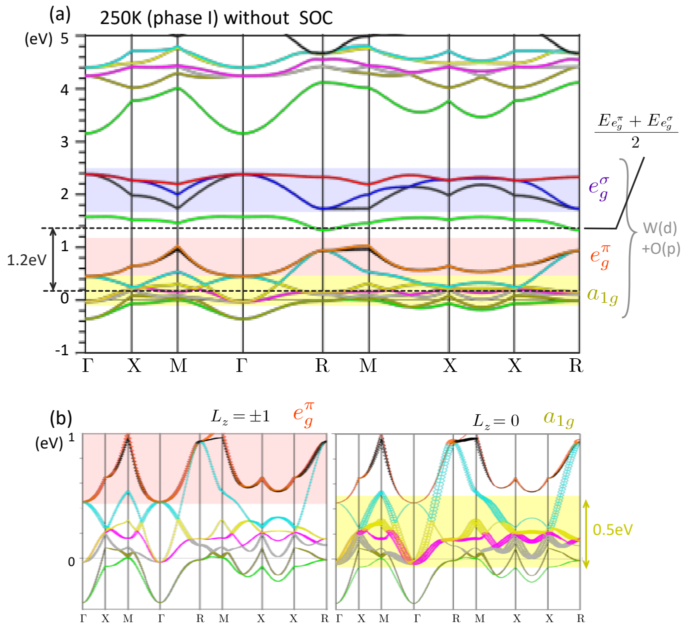

Supplementary Figure 2. Courtesy of Youichi Yamakawa [8]. Band structure of CsW_2O_6 at 250K without spin-orbit interaction obtained by the first-principles calculation (the same series of data as the right panel in Fig. 3 in Ref.[1].) (a) Band structures of t_{2g} levels, where we draw two broken lines which roughly gives the a_{1g} and e_g levels of the undistorted octahedron. The trigonal distortion and the effect from other metal ions split the e_g levels into bonding and antibonding levels, $e_g^{\pi/\sigma}$. (b) The same energy band as panel (a) where the local density of states from the $L_z^{\it eff}=\pm1$ and 0 are separately shown by the size of the symbols.

amplitude of local density of states of $L_z^{\it eff}=\pm1$ and 0 orbitals in Supplementary Figure 2(b); the larger symbol indicate the larger weight from these orbitals. By comparing this band structure with the one in Fig. 1**b**(main text) at $\lambda/t=0$, the band width $8t$ is evaluated as ~ 0.5eV, which gives $t\sim 0.06$.

We also set the bare a_{1g} level to approximately at around the flat dispersion at R-M line, as indicated by a broken line in Supplementary Figure 2(a). The bare e_g level that lies in the middle of the e_g^π and e_g^σ bands is also shown in broken line. The energy difference between these two broken lines is evaluated from Eq.(S5) as $(E_{e_g^\sigma}+E_{e_g^\pi})/2-E_{a_{1g}}=\frac{3}{2}a_4-\frac{5}{6}a_2\sim 1.2$ eV. We also see that $10Dq\sim$ 2eV from the difference between the E_g^σ and E_{a1g} levels. Combining these two relationships with $a_4=0.321$ from $\theta=55.71°$, we are able to determine $a_2=-0.142$ and accordingly, $\Delta_1=0.115(10Dq)=0.23$ eV. Using these values, we evaluate back the difference between t_{2g} and e_g^σ orbitals and obtain $E_{e_g^\sigma}-(E_{a_{1g}}+2E_{e_g^\pi})/3=1.01(10Dq)$, which is consistent with the definition of $10Dq$.

C. Slater-Koster parameters

We relate the transfer integrals t and λ of our Hamiltonian Eq.(1) in the main text with the microscopic parameter for CsW_2O_6. Here, in addition to the E_2 doublet which corresponds to CsW_2O_6, we also consider the E_3 doublet for comparison; this doublet shares the same constituents with the E_2 doublet, and Eq.(1) based on this E_3 doublet serves as an effective Hamiltonian for Iridates. We see that the model parameters t and λ differ much between the two doublets.

We specifically choose to evaluate the transfer integrals between site-4 $(x,y,z)=(3/8,3/8,3/8)$ and site-7 $(5/8,5/8,3/8)$ in Fig.1**a** in the main text without the loss of generality. Let us define three different coordinates; (x_0,y_0,z_0) is obtained by rotating the global Cartesian coordinate by $\pi/4$ about the z-axis. Local coordinate (x',y',z') for site-4 where z' serves as a C_3-rotational axis of the oxygen octahedra with $y'=y_0$, and (x'',y'',z'') for site-7 where z'' serves as a C_3-rotational axis with $y''=y_0$.

We have the relationships between the local basis set $\big(|xy\rangle,|yz\rangle,|zx\rangle,|x^2-y^2\rangle,|3z^2-r^2\rangle\big)$ and $(|a_{1g}\rangle,|e_{g1}^\pi\rangle,|e_{g2}^\pi\rangle)$ in Eq.(S4) and those with E_2 and E_3 doublets in Eq.(S8). The evaluation is given in three steps; we first obtain the Slater-Koster parameters[9] between the two sets of $\big(|x_0y_0\rangle,|y_0z_0\rangle,|z_0x_0\rangle,|x_0^2-y_0^2\rangle,|3z_0^2-r_0^2\rangle\big)$ on site-4 and site-7, given in the 5×5 matrices T_0^{oxy} and T_0^{dd} which are the oxygen mediated hopping and direct d-d hoppings, respectively. Then, the matrices are transformed from the representation of the $x_0y_0z_0$-axes to those between the local coordinates of site-4 and 7 using the rotation and mirror operation. Then the local basis sets are each transformed to the local t_{2g}-triplets using Eq.(S4), which gives the matrices, $T^{oxy}_{\gamma'\gamma''}$ and $T^{dd}_{\gamma'\gamma''}$. There, we also perform the same types of rotation and mirror from (x_0,y_0,z_0)-ones to the local axes to the spin-momentum axis as we did for the orbital angular momentum. The spin and orbital basis for each local coordinates with finite SOC is constructed using Eq.(S8), which gives the 2×2 matrix element for each doublet. There, we finally obtain the relationships between the values of t and λ to the Slater-Koster and other material parameters.

Direct and indirect hopping matrices. The direct d-d hoppings between site-4 and site-7 are given for $\big(|x_0y_0\rangle,|y_0z_0\rangle,|z_0x_0\rangle,|x_0^2-y_0^2\rangle,|3z_0^2-r_0^2\rangle\big)$ orbitals as

$$T_0^{dd}=\begin{pmatrix} v_{dd\pi} & & & & \\ & v_{dd\delta} & & & \\ & & v_{dd\pi} & & \\ & & & \frac{3v_{dd\sigma}+v_{dd\delta}}{4} & -\frac{\sqrt{3}(v_{dd\sigma}-v_{dd\delta})}{4} \\ & & & -\frac{\sqrt{3}(v_{dd\sigma}-v_{dd\delta})}{4} & \frac{v_{dd\sigma}+3v_{dd\delta}}{4} \end{pmatrix}, \tag{S10}$$

where $v_{dd\sigma}$, $v_{dd\pi}$, $v_{dd\delta}$, etc. are Slater-Koster parameters given in Ref.[9].

For the oxygen-mediated hoppings, we first consider

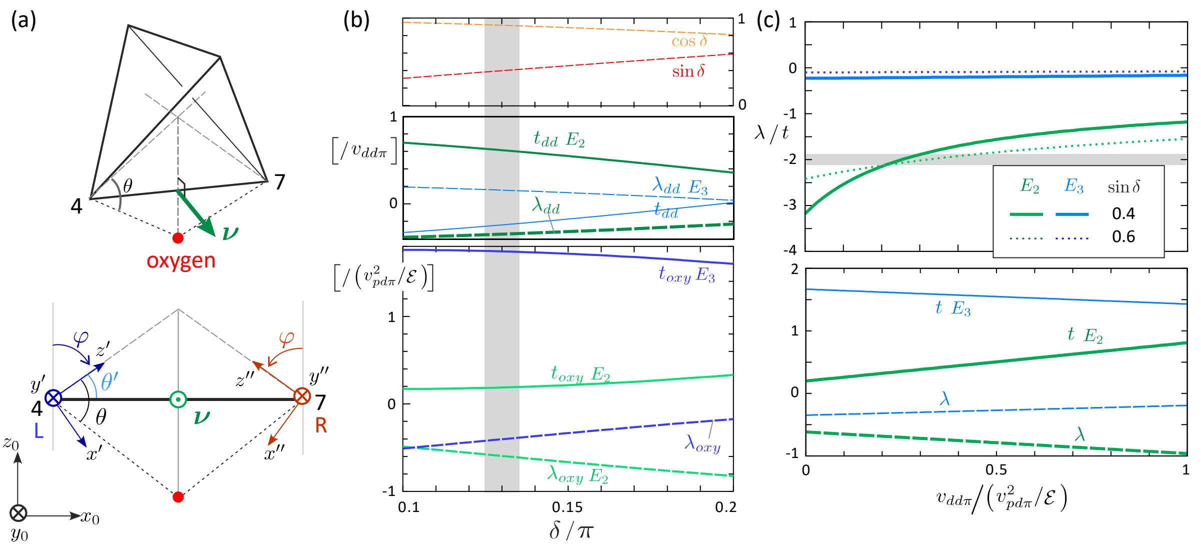

Supplementary Figure 3. (a) Site-4 and 7 used to miscoscopically evaluate the transfer integrals t and λ of the model. We adopt the $x_0y_0z_0$-axis as a common axis and rotate it by φ to construct the local coordinate which have z' and z''-axes as C_3 rotation axis of the trigonal symmetry. (b) Transfer integrals (t_{dd}, λ_{dd}) in unit of $v_{dd\pi}$ and (t_{oxy}, λ_{oxy}) in unit of $(v_{pd\pi}^2/\mathcal{E})$ originating from the direct dd-hopping and oxygen mediated hopping, respectively. The results are given separately for E_2 and E_3 doublets when varying δ in Eq.(S8). (c) Ratio of λ/t and t, λ when varying the ratio of Slater-Koster parameters taken as units for dd- and oxygen mediated hoppings, $x = v_{dd\pi}/(v_{pd\pi}^2/\mathcal{E})$.

the Slater-Koster parameter between p-oribals and d-orbitals given by a 3×5 matrix $V_{od;i}, (i = 4, 7)$ whose elements are

$$\begin{aligned}
(x|zx) &= \sqrt{3}\, c_t^2 s_t\, v_{pd\sigma} + s_t(1-2c_t^2)\, v_{pd\pi},\\
(x|x^2-y^2) &= \mp\frac{\sqrt{3}}{2} c_t^3\, v_{pd\sigma} \mp c_t s_t^2\, v_{pd\pi},\\
(x|3z^2-r^2) &= \mp c_t(s_t^2 - \frac{c_t^2}{2}) v_{pd\sigma} \pm \sqrt{3} c_t s_t^2\, v_{pd\pi},\\
(y|xy) &= \mp c_t\, v_{pd\pi},\\
(y|yz) &= s_t\, v_{pd\pi},\\
(z|zx) &= \mp\sqrt{3} s_t^2 c_t v_{pd\sigma} \mp c_t^2(1-2s_t^2)\, v_{pd\pi},\\
(z|x^2-y^2) &= \frac{\sqrt{3}}{2} s_t c_t^2\, v_{pd\sigma} - s_t c_t^2\, v_{pd\pi},\\
(z|3z^2-r^2) &= s_t(s_t^2 - \frac{c_t^2}{2})\, v_{pd\sigma} + \sqrt{3} s_t c_t^2\, v_{pd\pi},
\end{aligned} \tag{S11}$$

where $s_t = \sin(\theta-\theta')$ and $c_t = \cos(\theta-\theta')$ with angles given in Supplementary Figure 3(a) and $-/+$ or $+/-$ are cases for site-4/7. We thus obtain $T_0^{oxy} = {}^tV_{od;4}V_{od;7}$.

Axes rotations. For site-7, we first mirror the $x_0y_0z_0$-coordinate about the y_0z_0-plane to make it right-handed, and by rotating $+\varphi = \pi/2 - \theta'$ about the y-axis for both site-4 and 7, we obtain the local axis $(x'y'z')$ and $(x''y''z'')$, respectively. Here $\cos\theta' = \sqrt{2}/\sqrt{3}$ and $\sin\theta' = 1/\sqrt{3}$ is the same as the one we used in the main text. The relationships between the d-orbital basis defined on each orbital, $\mathbf{\Phi}^\dagger_{xyz} = (\langle xy|, \langle yz|, \langle zx|, \langle x^2-y^2|, \langle 3z^2-r^2|)$ is such that

$$\begin{aligned}
\mathbf{\Phi}_{x'y'z'} &= \mathcal{R}(\varphi)\ \ \mathbf{\Phi}_{x_0y_0z_0},\\
\mathbf{\Phi}_{x''y''z''} &= \mathcal{R}(\varphi)\mathcal{M}\ \ \mathbf{\Phi}_{x_0y_0z_0},
\end{aligned} \tag{S12}$$

where the mirror $\mathcal{M}$ converts the sign of the y and z-element of the angular momentum, and the rotation for the orbital angular momentum is given by

$$\mathcal{R}(\varphi) = \begin{pmatrix}
\cos\varphi & -\sin\varphi & & & \\
\sin\varphi & \cos\varphi & & & \\
 & & \cos^2\varphi - \sin^2\varphi & \sin\varphi\cos\varphi & -\sqrt{3}\sin\varphi\cos\varphi \\
 & & -\sin\varphi\cos\varphi & \frac{1+\cos^2\varphi}{2} & \frac{\sqrt{3}}{2}\sin^2\varphi \\
 & & \sqrt{3}\sin\varphi\cos\varphi & \frac{\sqrt{3}}{2}\sin^2\varphi & \cos^2\varphi - \frac{\sin^2\varphi}{2}
\end{pmatrix}$$

The corresponding SU(2) rotation of the coordinate of spinors by φ about the y-axis for site-4 and 7 is

$$D(\varphi) = \cos\frac{\varphi}{2}\hat{I} + i\sin\frac{\varphi}{2}\sigma_y. \tag{S13}$$

Transformation of matrices. We finally combine all the matrices and obtain the final form of the transfer integral matrix of doublets. Here, Eq.(S4) is represented by 3×5 matrix $A_{l\gamma}$ where l is the index for $(a_{1g}, e^\pi_{g1}, e^\pi_{g2})$ and γ is for $(xy, yz, zx, x^2-y^2, 3z^2-r^2)$. Once we obtain a 3×3 matrix for l and m pairs of t_{2g} orbitals from T_0^{oxy} by $A_{l\gamma}$, we include the denominator of the second order perturbation in the hopping process starting from orbital-l on site-7 and to oxygen p-orbital, and then to orbital-m on site-4, $-\frac{1}{2}(1/\mathcal{E}_l + 1/\mathcal{E}_m)$, where $\mathcal{E}_l, \mathcal{E}_m$ are the energy levels of oxygen p-orbitals measured from those of

d-orbitals(l,m). Setting $\mathcal{E}$ as their mean value, we find $\mathcal{E}_{a1g}=\mathcal{E}+2\Delta_1/3$ and $\mathcal{E}_{eg}=\mathcal{E}-\Delta_1/3$.

By introducing the spin up and down degrees of freedom, we obtain a matrix element $T_{l\sigma';m\sigma''}$ for $\big(|a_{1g;\uparrow}\rangle,|a_{1g;\downarrow}\rangle,|e^{\pi}_{g1;\uparrow}\rangle,|e^{\pi}_{g1;\downarrow}\rangle,|e^{\pi}_{g2;\uparrow}\rangle,|e^{\pi}_{g2;\downarrow}\rangle\big)$ of sites-4 and 7.

$$\begin{aligned} T^{dd}_{l\sigma';m\sigma''} &= \big(A_{l\gamma'}\mathcal{R}(\varphi)\,T^{dd}_0\mathcal{M}^{\dagger}\mathcal{R}(\varphi)^{\dagger}A^{*}_{m\gamma''}\big) \\ &\quad\otimes\big(D(\varphi)\mathcal{M}^{\dagger}D(\varphi)^{\dagger}\big). \\ T^{oxy}_{l\sigma';m\sigma''} &= \Big(A_{l\gamma'}\mathcal{R}(\varphi)T^{oxy}_0\mathcal{M}^{\dagger}\mathcal{R}(\varphi)^{\dagger}A^{*}_{m\gamma''}\Big)\frac{(-)}{2}\Big(\frac{1}{\mathcal{E}_l}+\frac{1}{\mathcal{E}_m}\Big) \\ &\quad\otimes\big(D(\varphi)\mathcal{M}^{\dagger}D(\varphi)^{\dagger}\big). \end{aligned} \tag{S14}$$

These 6×6 matrices are transformed by Eq.(S8) to 2×2 ones for the E_2 and E_3 doublets. Consistently with the symmetry argument, the form of matrices become $-t\hat{I}+i\sigma_y\sqrt{2}\lambda$, and we obtain (t_{dd},λ_{dd}) and (t_{oxy},λ_{oxy}) separately for the direct dd- and oxygen mediated hoppings.

In actually evaluating these parameters, we make use of the well-known relationshops of the Slater-Koster parameter, $v_{dd\sigma}=-1.5v_{dd\pi}<0$, $v_{dd\delta}=0$, $v_{pd\sigma}=-2.2v_{pd\pi}<0$, and apply $\theta=55.71^{\circ}$, $\Delta_1/\mathcal{E}=0\sim 0.2$. Then, (t_{dd},λ_{dd}) is obtained in unit of $v_{dd\pi}$ and (t_{oxy},λ_{oxy}) is obtained in unit of $v^2_{pd\pi}/\mathcal{E}$. The variation of paramters as functions of δ is shown in Supplementary Figure 3(b), where $(t_{dd},\lambda_{dd})=(0\sim 1,-0.5\sim 0)v_{dd\pi}$ and $(t_{oxy},\lambda_{oxy})=(0\sim 0.5,-1\sim -0.5)v^2_{pd\pi}/\mathcal{E}$ for E_2 doublets. The reason why λ_{oxy} is large and negative is that the hoppings between different species of d-orbitals $|zx\rangle$, $|x^2-y^2\rangle$ and $|3z^2-r^2\rangle$ mediated by the oxygen ions are antisymmetric and large, because p_x-orbitals that has the largest contribution extends toward site-4 and 7 with different signs.

As for E_3 doublets t_{dd} and λ_{dd} have overall opposite values from E_2. This can be understood from the δ-dependence of constituents of the orbitals in Eq.(S8). The t_{oxy} is large and positive, which is also because the sign of coefficients of $e^{\pi}_{g1/2}$ in Eq.(S8) is negative and the related elements of $T^{oxy}_{l\sigma';m\sigma''}$ between a_{1g} and $e^{\pi}_{g1/2}$ have opposite signs between two e_g's.

Since the oxygen p-orbitals are more extended than the d-orbitals, the major contribution to t and λ comes from the oxygen mediated ones [10,11]. In fact, several studies evaluating the model parameters of pyrochlore Iridates takes account only of the oxygen mediated ones[12], whereas the dd-hoppings have a small but finite contribution. Changing the ratio of these two varies the physical properties of the system[11]. We thus vary the ratio of the unit of these two contributions $x=v_{dd\pi}/(v^2_{pd\pi}/\mathcal{E})$ by taking $v^2_{pd\pi}/\mathcal{E}=1$ a unit and obtain $t=t_{oxy}+t_{dd}x$ and $\lambda=\lambda_{oxy}+\lambda_{dd}x$ as functions of $x=[0:1]$. As shown in Supplementary Figure 3(c), the large negative λ for E_2 doublet is obtained when the oxygen mediated ones is dominant, which gives large negative λ/t for all x. One sees that $\lambda/t=-2$ which yields a flat band can be quite reasonably realized for both variations of δ as well as x. For CsW_2O_6, we expect $\sin\delta\sim 0.4$ from Supplementary A.

The E_3 doublet has different tendency. Since λ_{dd} and t_{dd} are both much smaller than those of E_2, the x-dependence is very small, and λ/t is also very small. We have shown that E_2 and E_3 have different tendencies. However, this does not necessarily mean that the Iridates have small λ/t because we use the other parameter values for the CsW_2O_6. The details of parameter values for actual Iridates are not clarified.

We thus show that our model Hamiltonian Eq.(1) based on the E_2 doublet can realize a flat band by taking the parameter range consistent with the microscopic information on the materials. The E_3 doublet which is discussed in the context of Iridates, have an opposite tendency of model parameters with that of the E_2 doublet.

D. Noninteracting band structures

We plot in Supplementary Figure 4 a series of noninteracting band structures of $\mathcal{H}_{kin}$ in Eq.(1) in the main text, obtained by varying λ/t. These figures are to be compared with those of Fig. 1**b**. The flat bands at $\lambda/t=0$ and -2 become nearly flat when the parameters are off these values. Particularly when $\lambda/t<-2$, the nearly flat bands are gapped and for half-filling, become a topological insulator.

E. Details of the mean-field solution

We examined the competition between the metallic solution with uniform charge density $\langle n_{j\alpha}\rangle\sim 0.25$ ($\alpha=\uparrow,\downarrow$), 3-*in*-1-*out* solution with three charge rich sites located on the hyper-kagome site $\langle n_{j\alpha}\rangle\sim 1/3$, and 2-*in*-2-*out* solution having two charge rich sites $\langle n_{j\alpha}\rangle\sim 0.35$ and two charge poor sites $\langle n_{j\alpha}\rangle\sim 0.15$ per tetrahedron. The obtained energy per site E/N at $U/t=5$ are plotted for these solutions in Supplementary Figure 5(a) and 5(b). Particularly, the uniform metallic solution follows $E_{uniform}/tN=e_{pyro}/t+U/16t+3V/4t$, obtained by hand where e_{pyro} is the band energy of the noninteracting pyrochlore lattice at $U=V=0$ which is shown together in lines to compare with the mean field data points. The 3-*in*-1-*out* and 2-*in*-2-*out* solutions also show clear linear V/t dependences which are fitted by the following functions,

$$E/(tN)=e_{\phi}/t+a_{\phi}V/t,\quad \phi=\mathit{3in1out},\ \mathit{2in2out}. \tag{S15}$$

These coefficients are shown in Supplementary Figure 5(c). Here, the notable feature is that the effective noninteracting band energies of 2-*in*-2-*out* state, e_{2in2out} resembles those of the band energy of the hyperkagome lattice rather than the stripe solution. Also the V/t dependence of a_{3in1out} goes to 2/3 which is expected for

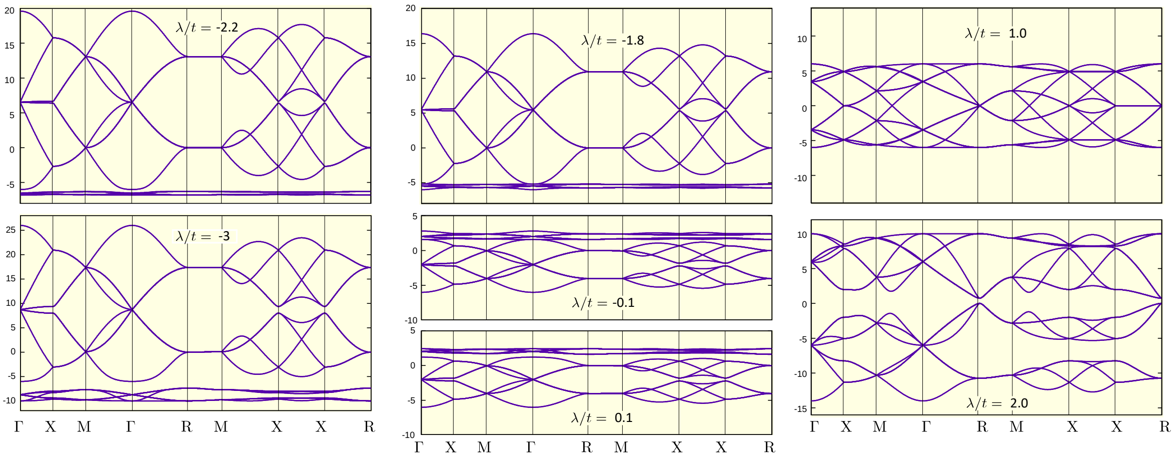

Supplementary Figure 4. Noninteracting energy bands of Eq.(1) $\mathcal{H}_{kin}$ for several choices of λ/t. The energy unit is taken as $t = 1$.

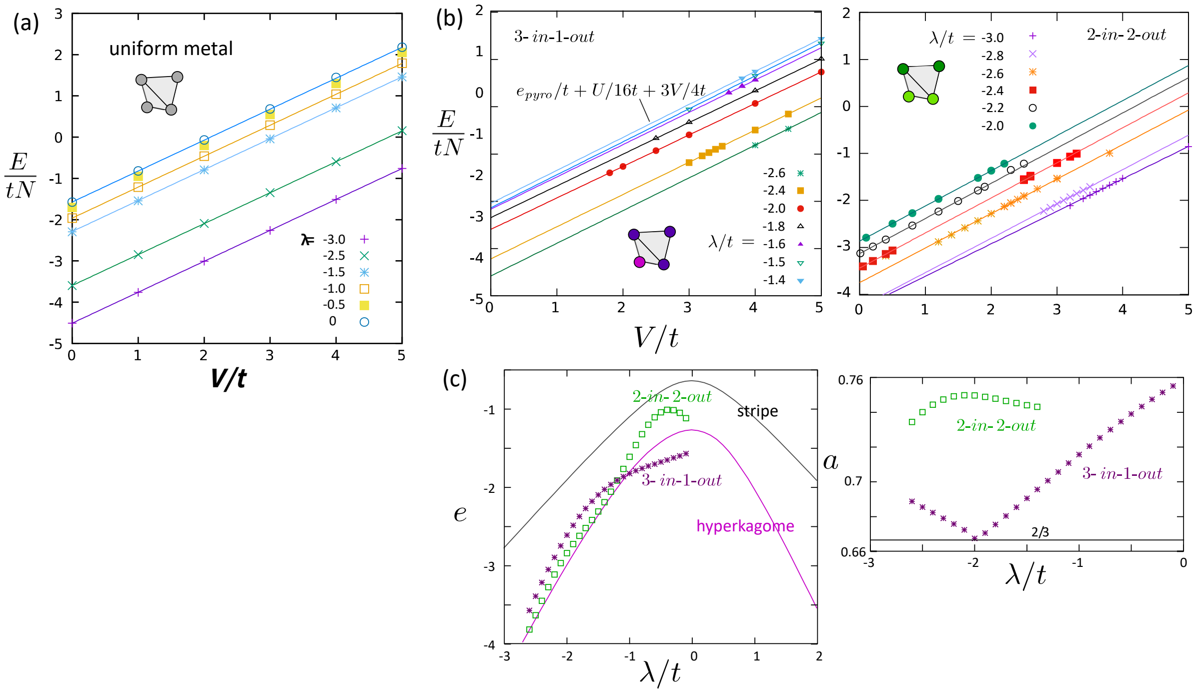

Supplementary Figure 5. (a) Energy per site of the uniform metallic mean field solution, which follows $E/N = e_{pyro}/t + U/16t + 3V/4t$. Here, e_{pyro} as a function of λ/t is given in Fig. 1 in the main text. (b) Energy per site of the 3-*in*-1-*out* and 2-*in*-2-*out* solutions, fitted via Eq.(S15). (c) Results of fitting given in panel (b), e_ϕ and a_ϕ, together with the noninteracting band energy of the hyperkagome and stripe states in solid lines for comparison.

the perfect flat band solution where we have 2/3 charges per triangular sites and 3/2 bonds per site which gives $(2/3)^2 \times (3/2) = 2/3$. Although this value linearly increases off this value, the mean-field solution is found to work very well to describe the 3-*in*-1-*out* flat band state since the charge density does not change much over a wide parameter range $-2.2 \le \lambda/t \le -1.8$.

F. Spinor line graph theory for the kagome lattice and some comments

We consider the Hamiltonian with transfer integral t and spin-orbit interaction λ,

$$\mathcal{H}_{kin} = \sum_{\langle i,j\rangle} \sum_{\alpha,\beta} \big(-t\delta_{\alpha\beta} c^\dagger_{i\alpha} c_{j\beta} + i\lambda c^\dagger_{i\alpha} (\boldsymbol{\nu}_{ij} \cdot \boldsymbol{\sigma})_{\alpha\beta} c_{j\beta}\big) + \text{h.c.}, \tag{S16}$$

where we set $t = 1$ and take

$$\boldsymbol{\nu}_{ij} = \frac{\boldsymbol{b}_{ij} \times \boldsymbol{d}_{ij}}{|\boldsymbol{b}_{ij} \times \boldsymbol{d}_{ij}|}, \tag{S17}$$

where notice that the factor differs by $\sqrt{2}$ from that of the main text for pyrochlore lattice. For the kagome lattice we find $\boldsymbol{\nu}_{ij} = (0, 0, \pm 1)$. We consider N_c unit cells where each unit cell includes three sites. The dual lattice is the honeycomb lattice with two sites per cell given in open red circles in Supplementary Figure 6(a). From the conventional line graph theory, the kagome band for $\lambda = 0$ has a flat band at the top level, $+2t$; $2N_c \times 3N_c$ matrix T_{DO} has two 1's on each column, which gives $T_{OD}T_{DO} = 2\hat{I} - \hat{H}_{\text{kagome}}/t$. The kernel $\{\boldsymbol{\varphi}\}$ which fulfills $T_{DO}\boldsymbol{\varphi} = \boldsymbol{0}$ has the dimension $3N_c - 2N_c = N_c$, and this gives a single flat band.

The extension to $\lambda \neq 0$ requires the elements of the above matrix to be replaced by twice the large one as

$$(\tilde{T}_{OD})_{ic_j} = \begin{cases} -i(\boldsymbol{r}_{ic_j} \cdot \boldsymbol{\sigma}) & \text{(connected)} \\ 0 & \text{(otherwise)} \end{cases} \tag{S18}$$

with $\boldsymbol{r}_{1c_1} = (-\sqrt{3/2}, -1/\sqrt{2}, 0)$, $\boldsymbol{r}_{2c_1} = (\sqrt{3/2}, -1/\sqrt{2}, 0)$ and $\boldsymbol{r}_{3c_1} = (0, \sqrt{2}, 0)$ shown in Supplementary Figure 6(a). Since $(-i\boldsymbol{r}_{1c_1} \cdot \boldsymbol{\sigma}) \cdot (+i\boldsymbol{r}_{2c_1} \cdot \boldsymbol{\sigma}) = -\hat{I} + \sqrt{3}i\sigma_z$ and $(-i\boldsymbol{r}_{1c_1} \cdot \boldsymbol{\sigma}) \cdot (+i\boldsymbol{r}_{1c_1} \cdot \boldsymbol{\sigma}) = 2\hat{I}$, we obtain the relationship

$$\tilde{T}_{OD}\tilde{T}_{DO} = 4\hat{I} + \hat{H}_{\text{kagome}}(\lambda/t = -\sqrt{3})/t.$$

Since the sign of λ does not change the nature of the system, we find the SOC flat band at $-4t$ when $\lambda/t = \pm\sqrt{3}$, which is demonstrated in Fig. 2 in the main text.

The SOC-induced flat band of the hyperkagome lattice has the construction almost the same as the pyrochlore one. One only needs to deplete 1/4 of the lattice sites and the same formulation applies.

We also show in Supplementary Figure 7(a)-(c) the examples of lattices where the present spinor line graph is difficult to apply. In these cases, the angles formed by the two $\boldsymbol{r}_{iC_n}$ vectors take $\theta/2 = \pi/2$, which requires $t = 0$ along the bonds while keeping $\lambda \neq 0$, when the flat band is realized. Also, the edge shared lattices cannot have the SOC pointing perpendicular to the two-dimensional plane. The next nearest neighbor SOC, fulfilling $\lambda = \sqrt{3}t$ can be realized on the honeycomb lattice in Supplementary Figure 7(c), but at the same time, $\lambda = \sqrt{3/2}t$ is required along the honeycomb bonds which is not possible, since $\lambda = 0$.

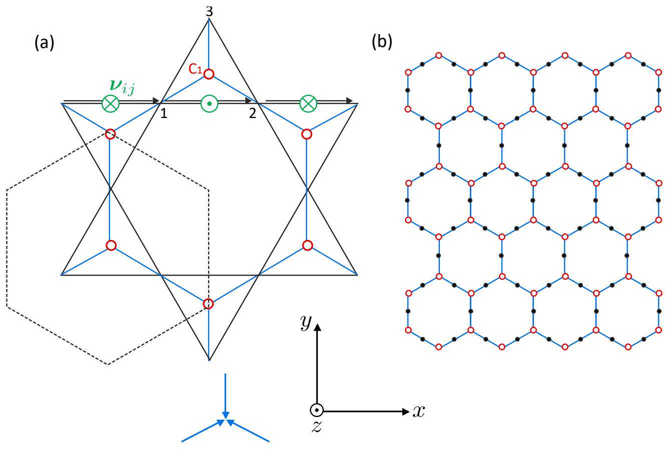

Supplementary Figure 6. (a) Kagome lattice and (b) the bipartite graph consisting of the dual honeycomb lattice(red open circle) and the kagome lattice (bullet) sites.

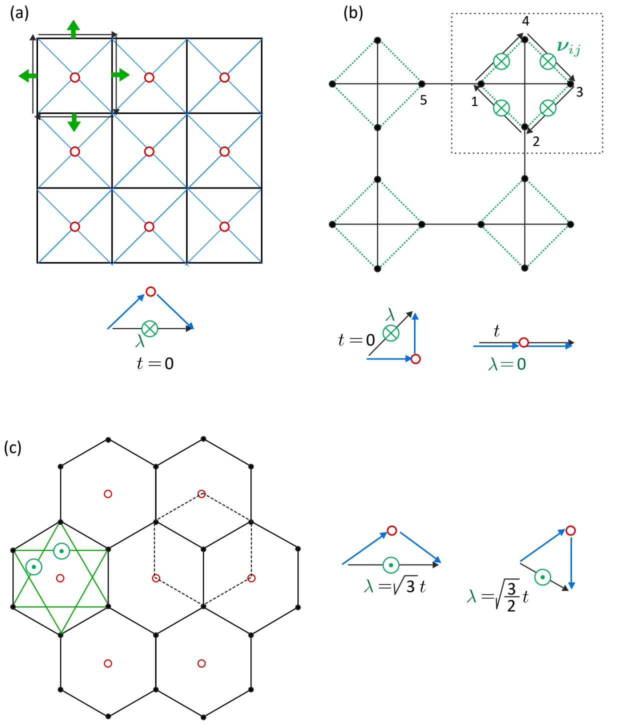

Supplementary Figure 7. Example of lattices which the SOC flat band is not easy to realize. (a) Square lattice, (b) Square-octagon lattice, and (c) honeycomb lattice. Lower panels are the constructions of $\boldsymbol{r}_{iC_n} \to \boldsymbol{r}_{jC_n}$ which gives the necessary condition for λ/t about the realization of the flat band. For $\theta/2 = \pi/2$ in cases like (a) and (b), $t = 0$ is required which is rather unrealistic. For the honeycomb lattice, the SOC along the nearest neighbor honeycomb bonds are usually zero, which does not fulfill the condition $\lambda = \sqrt{3/2}t$ in the right panel.

G. Construction of many body flat band state

1. one-body wave function

We show the details of how to construct a many-body wave function for the SOC flat band on the pyrochlore lattice. We first consider the one body wave functions

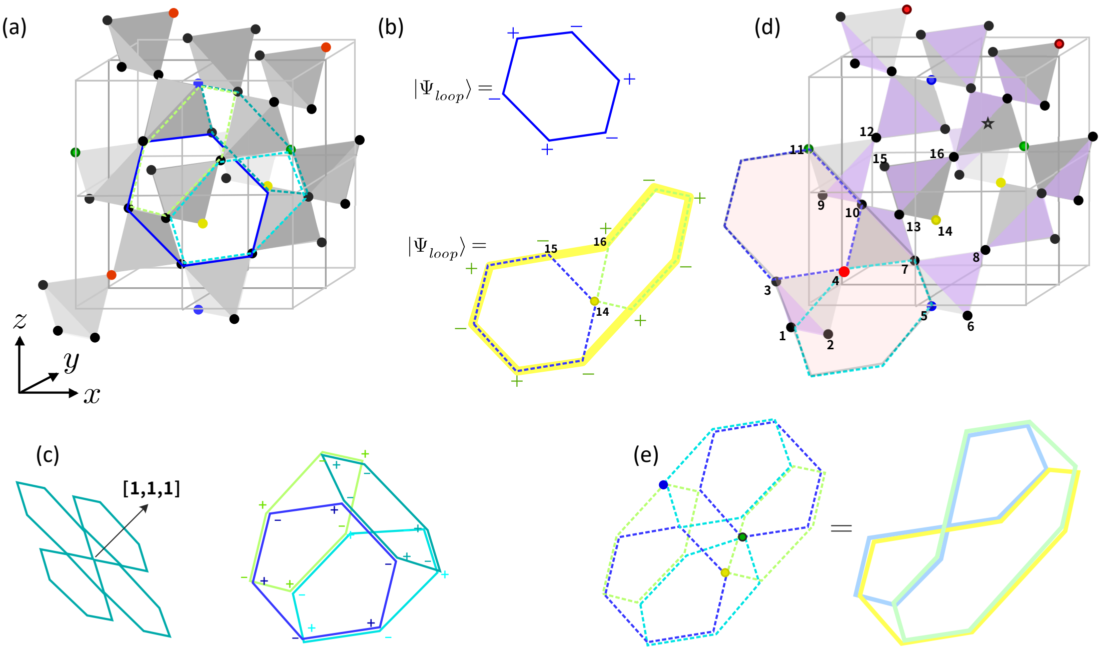

Supplementary Figure 8. (a) One-body wave functions consisting of six-site called loop-6. (b) Examples of loop-6 and loop-10 wave funtions. (c) Kagome plane normal to the $[1, 1, 1]$ direction consisting of corner shared hexagons, and four such hexagons sharing the edges, whose loop-6's are not independent. (d) Example of loop-10 wave function centered by site-4(red bullet) which is excluded in constructing a hyper-kagome structure. (e) Combination of three adjacent loop-10, where each loop-10 consists of two hexagons sharing blue/green/yellow sites which are to be vacant(non-hyper-kagome sites). These three loop-10's are not independent of each other.

satisfying the condition for the destructive interference, Eq.(7) in the main text. The hopping of electrons between the pyrochlore lattice points is equivalent to the indirect hoppings via the diamond lattice point. It is required that at each diamond lattice site, the summation of the hopping amplitudes from the neighboring pyrochlore sites is zero. The one body eigenstate is written in the general form as

$$|\varphi\rangle = \sum_{j=1}^{16N_c} (\varphi_{j\uparrow} c^\dagger_{j\uparrow} + \varphi_{j\downarrow} c^\dagger_{j\downarrow}) |0\rangle , \tag{S19}$$

and the condition Eq.(7) is written as;

$$\sum_{j\in n} (-i\boldsymbol{r}_{C_n j} \cdot \boldsymbol{\sigma}) \begin{pmatrix} \varphi_{j\uparrow} \\ \varphi_{j\downarrow} \end{pmatrix} = 0, \tag{S20}$$

where the sum is taken over the pyrochlore lattice points adjacent to C_n. We have four independent axes $\boldsymbol{r}_{C_n j}$ which are given for a 1-2-3-4 tetrahedron as $\boldsymbol{r}_{C_1 1} = (1, -1, 1)$, $\boldsymbol{r}_{C_1 2} = (-1, 1, 1)$, $\boldsymbol{r}_{C_1 3} = (1, 1, -1)$, and $\boldsymbol{r}_{C_1 4} = (-1, -1, -1)$. We determine the coefficients $\varphi_{j\sigma}$ by rotating the fictitious spinor on C_n, written as $\boldsymbol{\chi}_n =^t (u, d)$ about $\boldsymbol{r}_{jC_n}$ by an angle π:

$$\begin{pmatrix} \varphi_{j\uparrow} \\ \varphi_{j\downarrow} \end{pmatrix} = -i\eta_j (\boldsymbol{r}_{jC_n} \cdot \boldsymbol{\sigma}) \boldsymbol{\chi}_n \tag{S21}$$

where η_j is the additional phase factor. Since

$$\sum_{j\in n} (-i\boldsymbol{r}_{jC_n} \cdot \boldsymbol{\sigma}) \begin{pmatrix} \varphi_{j\uparrow} \\ \varphi_{j\downarrow} \end{pmatrix} = \sum_{j\in n} 3\eta_j \boldsymbol{\chi}_n, \tag{S22}$$

this state meets the condition by choosing the proper phase factors that satisfy $\sum_{j\in n} \eta_j = 0$.

For example, one can construct a closed loop consisting of six sites as shown in Supplementary Figures 8(a) and (b) where along that loop the phase factors can be chosen as $+1, -1, +1, \cdots$. Since the two adjacent sites on that loop belong to the same tetrahedron, the condition Eq.(S22) is easily satisfied. The actual form of the one-body wave function is obtained by setting e.g. $\boldsymbol{\chi}_n =^t (1, 0)$ for all C_n connected to the loop, and the direction of the spins is fixed following the π-rotation from this fictitious spinor. If the number of electrons is much smaller than N, one can construct such loops located apart over a few lattice spacing from each other and put the electrons, which gives $\langle \mathcal{H}_I \rangle = 0$. However, in the present case, half of the flat band is filled and the electrons are densely packed. Therefore, we need to explicitly consider the number of independent one-body flat bands and using them as building blocks, construct a many-body wave function.

The shortest loop consists of six sites/bonds which we call loop-6, and one can have four species of loop-6 which are normal to four directions, $[1, \pm 1, \pm 1]$, respectively.

They belong to four different kagome layers normal to these vectors (see Supplementary Figures 8(c) for those normal to $[1, 1, 1]$). For each kagome layer, one finds four independent loop-6's included in the extended unit cell consisting of 16 sites. This seemingly gives us 16 different loop-6 per unit cell. However, among them only half are independent; as shown in Supplementary Figures 8(c), one can choose adjacent four loop-6 which share half of their edges. Then, by assigning ± 1 weight in constructing the wave function (phases can be gauged out) these four cancel out and they are no longer independent of each other. We have eight such constraints per unit cell, and the number of independent loop-6 is reduced to eight. Considering the spin degrees of freedom $\boldsymbol{\chi}_n = {}^t(1, 0)$ and ${}^t(0, 1)$, we find sixteen loop-6 per cell.

2. *trimerized many-body wave function*

In CsW_2O_6, we find a trimerized charge-ordered state with one of the four sites of the tetrahedron being perfectly vacant. The sites that are vacant are colored in red(site-4)/blue(5)/green(11)/yellow(14) in the unit cell as shown in Supplementary Figure 8(d). The resultant hyper-kagome structure consists of corner-shared triangles avoiding these sites. In constructing a one-body flat band wave function that fulfills Eq.(S20) but avoiding these vacant sites, we need to extend the length of the loop to ten by connecting two hexagons sharing these colored vacant sites; for example, this loop-10 around the red site-4 is shown in Supplementary Figure 8(d). For each vacant site, we have three hexagons that are not shared with other vacant sites. There can be at most two independent loop-10 out of them. Since we have four vacant sites the 8($\times$2 with spin degrees of freedom) independent loop-10 may be expected. However, in the same manner as loop-6, three loop-10's sharing part of their edges are not independent, as shown in Supplementary Figure 8(e). This will again reduce the number of independent loop-10 to 4($\times$2) per unit cell.

In considering a large but finite system with periodic boundary, one needs to care about the extra wave function that originates from a band touching. Among $4N_c \times 2$ different loop-10, if we choose the periodic boundary, there can be another constraint that collecting the whole $4N_c \times 2$ such wave functions make them no longer independent. This will reduce the number of independent loop-10 to be $(4N_c - 1) \times 2$. However, there are additionally large loops around the two sides of the periodic boundaries that also form a loop wave function. Therefore, adding these two will give totally $(4N_c + 1) \times 2$ independent wave functions.

Once a whole set of one-body wave function is obtained, one can in principle construct a many-body flat band wave function in a product form of the one-body flat band state as

$$|\Psi_{3in1out}\rangle \propto \prod_{n,\sigma} \hat{\psi}^{10}_{n,\sigma} |0\rangle ,$$

$$\hat{\psi}^{10}_{n,\sigma} = \frac{1}{\sqrt{30}} \sum_{j \in \mathrm{loop}-10} (\varphi_{j\uparrow} c^\dagger_{j\uparrow} + \varphi_{j\downarrow} c^\dagger_{j\downarrow}), \qquad \text{(S23)}$$

where $\varphi_{j,\sigma}$ fulfills the aforementioned constraint with two choices of $\boldsymbol{\chi}_n = {}^t(1, 0)$ and ${}^t(0, 1)$ (this choice is indexed as σ of $\hat{\psi}^{10}_{n,\sigma}$). This form is general, whereas in a practical calculation if we use the regular copies of loop-10 for all unit cells, they can easily give zero due to the above mentioned constraint, and we need to irregularly choose loop-10 while still keeping the distribution charges to be close to uniform on a hyper-kagome lattice to minimize the loss of interaction V. Since we need to host $8N_c$ electrons, this flat band wave function consisting of loop-10 is fully occupied. For this reason, the overall energy gain of having the flat band wave function is not much different from the mean-field evaluation, although precisely evaluating $\langle H_I \rangle$ is practically difficult due to the abovementioned nontrivial constraint.

---

* nakai-hiroki3510@g.ecc.u-tokyo.ac.jp

† chisa@phys.c.u-tokyo.ac.jp

[1] Y. Okamoto, H. Amano, N. Katayama, H. Sawa, K. Niki, R. Mitoka, H. Harima, T. Hasegawa, N. Ogita, Y. Tanaka, M. Takigawa, Y. Yokoyama, K. Takehana, Y. Imanaka, Y. Nakamura,H. Kishida and K. Takenaka, Nat. Comm. **11**, 3144 (2020).

[2] K. I. Kugel, D. I. Khomskii, A. O. Sboychakov, S. V. Streltsov, Phys. Rev. B **91**, 155125 (2015).

[3] D. Uematsu, H. Sagayama, T. Arima, J. J. Ishikawa, S. Nakatsuji, H. Takagi, M. Yoshida, J. Mizuki, and K. Ishii, Phys. Rev. B **92**, 094405 (2015).

[4] B. H. Kim, G. Khaliullin, and B. I. Min, Phys. Rev. B **89**, 081109(R) (2014).

[5] D. Mandrus, J. R. Thompson, R. Gaal, L. Forro, J. C. Bryan, B. C. Chakoumakos, L. M. Woods, B. C. Sales, R. S. Fishman, and V. Keppens Phys. Rev. B **63**, 195104 (2001).

[6] A. Uehara, H. Shinaoka, and Y. Motome, Physics Procedia, **75**, 495 (2015).

[7] T. Takayama, J. Chaloupka, A. Smerald, G. Khaliullin and H. Takagi, J. Phys. Soc. Jpn. **90**, 062001 (2021).

[8] Youichi Yamakawa, private communications.

[9] J. C. Slater and G. F. Koster, Phys. Rev. **94**, 1948 (1954).

[10] W. Witczak-Krempa, A. Go, and Y. B. Kim, Phys. Rev. B **87**, 155101 (2013).

[11] W. Witczak-Krempa and Y. B. Kim, Phys. Rev. B **85**, 045124 (2012).

[12] D. Pesin and L. Balents, Nat. Phys. **6**, 376 (2010).
